# How antimalarial drug resistance affects post-treatment prophylaxis

**DOI:** 10.1186/1475-2875-7-9

**Published:** 2008-01-11

**Authors:** Nicholas J White

**Affiliations:** 1 Mahidol-Oxford Research Unit, Faculty of Tropical Medicine, Mahidol University, 420/6 Rajvithi Rd., Bangkok 10400, Thailand; 2 Centre for Clinical Vaccinology and Tropical Medicine, University of Oxford, Churchill Hospital, Oxford, OX3 7LJ, UK

## Abstract

Slowly eliminated antimalarial drugs suppress malaria reinfections for a period of time determined by the dose, the pharmacokinetic properties of the drug, and the susceptibility of the infecting parasites. This effect is called post-treatment prophylaxis (PTP). The clinical benefits of preventing recrudescence (reflecting treatment efficacy) compared with preventing reinfection (reflecting PTP) need further assessment. Antimalarial drug resistance shortens PTP. While blood concentrations are in the terminal elimination phase, the degree of shortening may be estimated from measurements of in-vitro susceptibility and the terminal elimination half-life. More information is needed on PTP following intermittent preventive treatments, and on the relationship between the duration of PTP and immunity, so that policy recommendations can have a firmer evidence base.

## Background

In areas of intense malaria transmission reinfection following antimalarial treatment is inevitable. Antimalarial drug treatments with slowly eliminated drugs provide a variable period, after they have cleared the initial infection, during which subsequent reinfection is suppressed by residual levels of the antimalarial drug. This effect is called post-treatment prophylaxis or PTP [1,2]. PTP can range in duration from months, with very slowly eliminated compounds such as piperaquine and chloroquine, to none at all with rapidly eliminated drugs such as the artemisinin derivatives. PTP should provide additional benefit, above that conferred by effective cure of a symptomatic infection, by allowing a longer disease-free interval for clinical and haematological recovery. The benefits of preventing reinfection depend on the transmission intensity and thus the frequency of infection. It has been argued that preventing reinfection is as important as preventing recrudescence, although this remains to be proved, both at an individual and population level. The two are obviously linked as both are measures of antimalarial efficacy. This individual benefit of longer PTP is balanced against a potential societal harm; the increased propensity of slowly eliminated antimalarials to select for resistance [3-5]. PTP is an important component of intermittent preventive treatment, (also sometimes referred to as intermittent presumptive treatment) in pregnancy (IPTp) and is probably the most important effect of intermittent preventive treatment in infancy (IPTi), and in other high risk sub-groups [1,6,7]. The dose and the pharmacokinetic properties of the drug affect the duration of PTP. For the prevalent malaria parasites, antimalarial drug resistance reduces the duration of PTP.

In practice, PTP is measured as the time from drug administration (either treatment of the first, usually symptomatic, infection or administration of IPT to an asymptomatic person) until the next infection is detectable on a blood smear. In areas of very intense malaria transmission (e.g one infected bite per person per day), this interval can be assessed in an individual, but at lower levels of transmission, where infections are acquired at a low frequency, there is a large stochastic element to the acquisition of infections, and so populations must be studied. Prevention of malaria in pregnancy has an additional component in that the pharmacokinetic properties of many drugs are altered in late pregnancy. Furthermore the *Plasmodium falciparum *parasites which establish in the placenta bind specifically to chondroitin sulphate A, and as these infections are considered to be a minority of natural infections, they may take longer to become established than systemic infections. Although IPTp has been widely introduced with sulphadoxine-pyrimethamine (SP), and there is increasing support for IPTi, there have not been sufficient studies to characterize the critical relationship between the duration of PTP for the candidate drugs and resistance to them to guide policy. Here, the relationship between the level of antimalarial drug resistance and the duration of PTP is reviewed from a pharmacological perspective, and suggestions provided for future investigations.

### Pharmacokinetic determinants

Malaria parasites infect red blood cells. To kill, or to inhibit the development of the intraerythrocytic parasite, the antimalarial drug must enter the infected red cell and attain a sufficient intraparasitic concentration. The biologically relevant component of antimalarial drug content in blood is the free plasma concentration (Cf), as this is thought to reflect best the concentrations to which the malarial parasite is exposed. Concentration gradients exist within the parasite, and there may be considerable accumulation within the food vacuole, but all depend on the concentration of antimalarial in plasma water. Cf is the product of the total plasma concentration (C) and the proportion of plasma drug in plasma water (i.e. unbound to plasma proteins) – the free fraction (f).

The concentration of drug in blood at any time is determined by several independent variables; the rate of absorption, the fraction of drug absorbed (the bioavailability), the apparent volume(s) of distribution, and the rates of distribution and clearance. Each of these may be affected by the disease process and the physiological changes occurring in pregnancy or infancy. All the antimalarial drugs are eliminated by a first order process, and in this review it is assumed that PTP still occurs during the terminal elimination phase of the drug. The calculations focus on this phase.

i.e. at any time (t) in this phase C f_t _= C f_0 _e^-kt^

where C f_0 _is the intercept back extrapolated to time = 0 for the terminal phase, and k is the first order terminal elimination rate constant

k = ln2/t_1/2 _where t_1/2 _= the terminal elimination half life.

Although essentially derivative, the terminal elimination half-life is a familiar concept to most clinicians and malariologists and so will be the main variable used in the following examples.

### Pharmacodynamic determinants

The level of resistance determines the antimalarial drug effect at any given drug concentration. Unfortunately it is not possible to extrapolate directly from in-vitro to in-vivo measurements of drug susceptibility, although clearly there is a relationship between the two.

If the minimum inhibitory concentration (MIC) is the lowest concentration inhibiting multiplication

i.e. the concentration at which parasite multiplication rate (PMR) = 1

then MIC in vivo = *f *MIC in vitro

Assuming this to be a linear function then

0.5 × MIC in vivo = 0.5 × *f *MIC in vitro

In the terminal elimination phase the time taken for drug concentrations to fall by half is then, by definition, one half-life.

**Figure 1:**
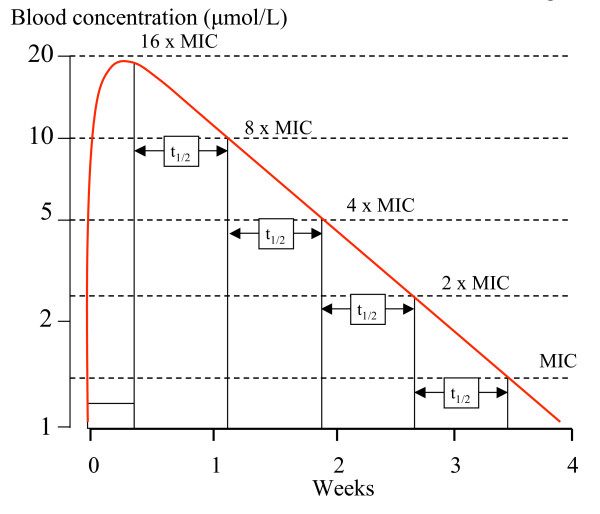
In this diagram a slowly eliminated drug given over three days has a single component (monoexponential) elimination phase with a half-life of 5.5 days. This corresponds with a one-compartment model. The vertical axis has a logarithmic scale. The in-vivo MIC is 1.25 μmol/L. Each doubling of MIC shortens the PTP by one half-life.

Put another way, provided that the MIC values remain below the concentrations which occur at the end of the distribution phase (i.e. the beginning of the terminal elimination phase), then resistance which results in a doubling of the MIC will shorten the duration of PTP by one half-life [1]. So during the terminal elimination phase, if the MIC increases N, fold the corresponding reduction in PTP duration measured in terms of half-lives is log_2 _N (log_2 _is the logarithm to the base 2) (Figure 1).

Immunity also inhibits parasite development and multiplication and thereby augments the effects of antimalarial drugs. This explains the greater efficacy of failing antimalarial drugs in areas of moderate to high malaria transmission in older children and adults. It also explains why IPT_p _with SP appears to provide benefit even in the face of significant resistance. In effect immunity moves the dose-response relationship to the left (i.e. the opposite direction to resistance). The calculations that follow assume either no immunity or a constant effect of immunity. If a drug has a single terminal elimination phase then there is a predictable limit to the possible effect of resistance on PTP. If the maximum duration of PTP with fully sensitive parasites is "P" half-lives then it is obviously not possible to shorten PTP by more then P half-lives. So, with the provisos above, there is no PTP once MICs have risen to the level where

**N = log**_2_**P × P**

For example if the maximum PTP is 16 half-lives (in fully sensitive parasites) then there is no PTP at all if the MICs rise more than 64 fold (64 = log_2_16 × 16).

Unfortunately, there are not very accurate estimates of in-vivo MIC, nor of PTP durations against sensitive parasites, although Watkins *et al *[8] have estimated a figure of approximately 60 days for fully sensitive *P. falciparum *following SP administration, and PTP is certainly more than a month for both chloroquine and mefloquine against fully sensitive *P. falciparum *and *Plasmodium vivax*.

**Figure 2:**
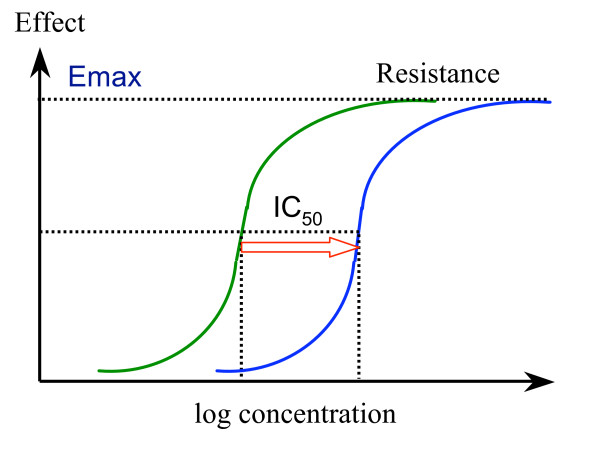
Worsening resistance; the antimalarial concentration-effect relationship moves to the right. In this example the shift is parallel. IC50 is the concentration giving 50% of maximum effect, and Emax is the maximum effect possible. Usually in-vitro susceptibility is assessed by growth inhibition, inhibition of uptake of ^3^H Hypoxanthine, or inhibition of formation of pLDH or PfHRP2.

The relationship between antimalarial drug concentration and inhibition of parasite growth is generally sigmoid (1) but the relationship between inhibition of growth and inhibition of parasite multiplication has not been well characterized. Nor is it clear how immunity affects this relationship. Resistance results in a shift to the right of the antimalarial concentration-growth inhibition (dose-response) curve. If the slope does not change then the respective positions on the curve also do not change (Figure 2).

Assuming a parallel shift in the dose response (concentration-effect) relationships and linear relationship between inhibition of growth and inhibition of multiplication, then, with these two important provisos, the change in IC_50 _with resistance is exactly equivalent to the change in MIC.

i.e. Δ IC_50 _= Δ MIC

For example, if there was an eight-fold increase in IC_50 _there would be a corresponding eight fold increase in MIC, and so, from equation (1), the duration of PTP would be reduced by log^2 ^8 – or three fold (Figure 1).

Untramelled parasite multiplication can theoretically achieve a multiplication rate equal to the mean number of viable merozoites per schizont at schizont rupture. In non-immune patients and volunteers multiplication rates averaging approximately 8–10/cycle have been observed (although rates over 20 have been documented in some individuals) [9,10]. The MIC is defined as the antimalarial drug concentration giving a net growth rate of 1, so the MIC is certainly not at the "bottom" of the concentration-effect curve. Inhibition of growth still occurs at sub-MIC concentrations. Thus, when plasma (or blood) concentrations of antimalarial drug have fallen below the MIC, parasite growth is possible, but the rate of parasite expansion is reduced because of residual drug effects. Fully efficient multiplication is possible only when concentrations have fallen to the bottom of the concentration-effect curve (figure 2).

The longer the t_1/2 _the longer is this period of sub-MIC residual suppression and the longer the interval from falling below MIC levels to the appearance of patent parasitaemia. If the resistant parasites have a fitness disadvantage conferring reduced multiplication, then the interval is further prolonged.

### Multiphasic elimination

**Figure 3:**
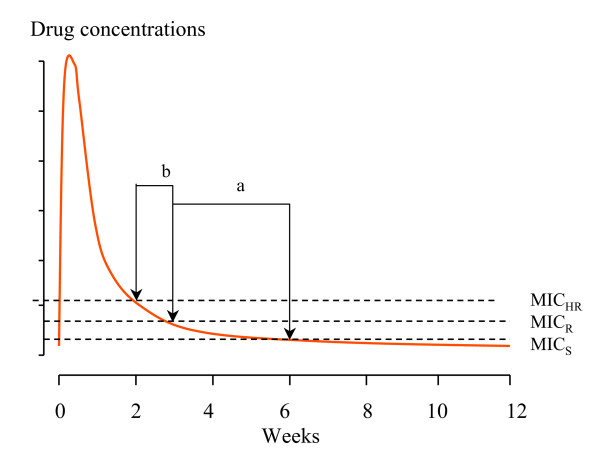
Three levels of antimalarial susceptibility for a slowly eliminated antimalarial with a multiphasic elimination profile (e.g chloroquine, piperaquine) are shown reflected by the respective minimum inhibitory concentrations (MIC); sensitive (S), resistant (R) and highly resistant (HR). Post treatment prophylaxis for sensitive parasites is six weeks. In this example increasing levels of resistance progressively shorten the PTP from six to three weeks (a) and then from three to two weeks (b).

Several of the antimalarial drugs (such as chloroquine, desethylamodiaquine, tafenoquine, pyronaridine, mefloquine, piperaquine) have complex pharmacokinetic properties as they bind extensively to tissues and have large total apparent volumes of distribution and multiphasic elimination profiles with a long terminal phase. These elimination profiles can be characterized by a series of exponential terms. For example, chloroquine has a terminal elimination half-life of one to two months. Piperaquine also has a very long terminal elimination half-life. If the duration of PTP is six weeks then it is obviously not possible to shorten this by more than one half life. With increasing resistance the concentrations required for inhibition of parasite growth rise above those seen in the terminal phase. The duration of PTP is determined then by distribution processes, and chloroquine becomes effectively a "shorter half-life drug". This results in a non-linear relationship between duration of PTP and the in-vivo MIC (Figure 3). A drug with a single terminal elimination phase characterised by a single exponential term is shown in Figure 1, and a multiphasic elimination profile in Figure 3.

### Population considerations

So far the hypothetical changes that would occur in an individual confronted with different parasites have been considered. But in reality a population of individuals, in whom pharmacokinetic properties often differ considerably, confront populations of malaria parasites with different drug susceptibilities. These two distributions are unrelated. The distribution of antimalarial pharmacokinetic variables is often approximately normal or log normal, whereas susceptibility distributions can be either continuous (e.g. often for quinolines and related drugs) or multimodal (antifolates). Following antimalarial treatment in a high transmission setting the most resistant parasites will, by definition, tend to establish themselves first in a subject as blood concentrations decline. This is the force that underlies selection of resistance in the elimination phase. It is also why examining parasites causing reinfections after treatment or intermittent prophylaxis with a slowly eliminated drug gives a false impression of the degree and prevalence of antimalarial drug resistance.

**Figure 4:**
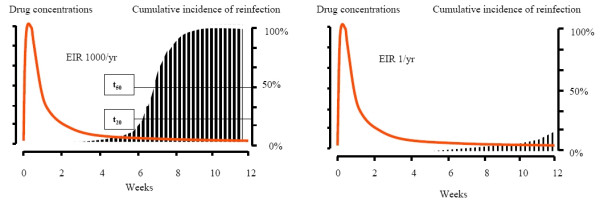
Post treatment prophylaxis following a slowly eliminated antimalarial such as chloroquine or piperaquine in a high transmission setting (left figure) where the EIR is 1000/year and in a low transmission setting where the EIR is 1/year. The drug concentrations are shown in red and the vertical bars represent cumulative incidence of reinfection; t_20 _and t_50 _are the times to reach a 20% and 50% cumulative incidence of reinfection.

**Figure 5:**
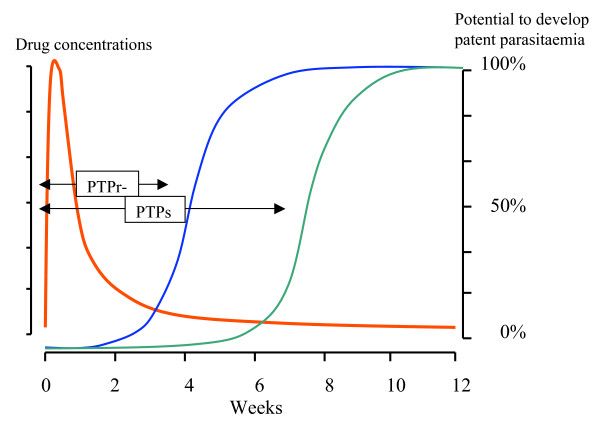
The figure refers to the example. The drug concentrations are shown in red, the cumulative incidence of reinfection with resistant parasites is shown in blue, and the cumulative incidence of reinfection with sensitive parasites is shown in green.

In a high transmission setting the cumulative probability of detecting parasitaemia following treatment will be determined by pharmacokinetic-pharmacodynamic (PK-PD) factors; it is a mirror of the population profile of antimalarial activity (a function of the population's elimination phase kinetics and the distribution and shapes of the parasite growth inhibition curves). In low transmission settings, this is more complex as the stochastic element determining the probability of being bitten by an infected mosquito is much larger. The average duration of PTP at a population level may be defined as the duration for which suppression of a certain proportion of reinfections is suppressed. In a high transmission setting this could be time to 20% (t_20_) or 50% (t_50_) prevalence of reinfection (Figure 4). In a low transmission setting this is more difficult to characterise. If there are different resistance genotypes prevalent then these values should ideally be defined for each genotype (as in the example and Figure 5).

### Example (Figure 5)

In parts of Tanzania, the EIR was 350 infectious bites per person per year (1/day). At one point the prevalence of the PfDHFR Asn108 mutation, conferring an approximate ten-fold reduction in susceptibility, among parasites was at one time 5% and the remaining parasites were "wild type". In the 1950s Pyrimethamine alone (t_1/2 _3 days) was widely used as treatment. The average PTP against parasites with the Asn108 mutation would be expected to be reduced by log_2 _10 × 3 = 10 days compared with that against sensitive parasites.

With one infection per day and a 5% prevalence of the PfDHFR Asn108 mutation there would therefore be a 50% probability of acquiring such an infection within a 10 day period. Thus, although the background prevalence of resistant parasites was 5% the proportion of resistant parasites causing the first reinfection would have been expected to be approximately 55%. It is important to note that finding an increased proportion of "resistant" parasites during the terminal elimination phase of a slowly eliminated drug is expected [11]. It is an indicator of the selective pressure, but many other factors also influence of degree of selection.

### Immunity

Immunity reduces the probability of an infection becoming patent. Immunity to malaria parasites is complex and poorly defined, but is generally unrelated to drug resistance. In malaria endemic areas therapeutic responses vary with age as young children have little or no immunity compared with older children and adults. Immunity results in better therapeutic responses for any level of resistance. Even in areas of low transmission cure rates for any failing drug (with pharmacokinetics which are not age dependent) are higher in adults than children. Intermittent preventive treatment with sulphadoxine-pyrimethamine in pregnancy appears still to be beneficial where 14-day failure rates in young children are as high as 40% [6]. Immunity, therefore, contributes to PTP, but the interaction between immunity and treatment responses has not been well characterized.

## Discussion

Antimalarial drug resistance shortens the period of post-treatment prophylaxis. This is evident from prospective therapeutic studies in both falciparum and vivax malaria. In infections with tropical "strains" of *P. vivax*, the first sign of chloroquine resistance is the appearance of parasitaemia within 28 days of starting chloroquine treatment [12]. This represents breakthrough of the first relapse (which normally becomes patent around three weeks after starting treatment), and is suppressed in chloroquine-sensitive *P.vivax *infections [2]. In falciparum malaria, reinfections occur earlier and earlier as resistance worsens. The changing pattern is determined by the unrelated distributions of host pharmacokinetics and parasite susceptibility. Post-treatment prophylaxis is an important component of treatment responses [13] and is probably the main component of IPT. Unfortunately, there are very few data on the duration of PTP following IPT. Obtaining this information is necessary in order to determine the relationship between PTP and important clinical end-points such as anaemia and birthweight, and thus the effects of resistance [14]. This would help considerably to rationalize IPT policy, as there are currently no guidelines on when to stop IPT in the face of increasing resistance. Conducting trials with clinical end-points, or even their surrogates (e.g. placental parasitaemia) requires very large sample sizes and, therefore, is both costly and time consuming. Importantly information on PTP is already available within prospective drug trials which have adequate durations of follow up (> 28 days), although it is usually not examined explicitly. PTP should be assessed in the relevant patient group as it is clearly affected by immunity, and the pharmacokinetic properties of the drug may well be altered in the target group. SP is the most widely used drug for IPT. The "standard" doses used have been extrapolated from studies in non-pregnant adults, yet we have only recently learned that the pharmacokinetic properties of SP are markedly altered and drug levels consequently lower in two of the main IPT target groups – pregnant women and young children aged 2–5 years [15,16]. Unfortunately there are still no data at all on the pharmacokinetic properties of SP in infancy.

Assessing PTP requires measurement of drug levels, although for drugs with terminal elimination half-lives of less than one week these levels have commonly fallen below levels of detection when the first reinfection occurs. In these cases, earlier measurement (e.g. the day 7 level) helps with interpretation [17]. Ideally studies would have both an individual pharmacokinetic assessment and a test of parasite susceptibility – but culturing the low density parasitaemias at first reinfection is often difficult. Fortunately molecular markers of resistance are now available for several of the antimalarial drugs-notably SP, and these can be assessed in samples from patients with low-density parasitaemias. Antimalarial drugs are a central component of malaria control, but they are threatened by increasing resistance [18]. Fortunately new drugs are becoming available. By characterizing the pharmacokinetic and pharmacodynamic properties of the antimalarial drugs in the appropriate patient groups, their use can be rationalised and optimised.

## Abbreviations

### Distribution phase

The phase following drug administration during which the drug distributes to and exchanges with the tissues. During this phase blood concentrations may fall faster than during the elimination phase.

### Elimination phase

Following distribution, the period during which the drug is being eliminated. This may have one or more phases. The last is the terminal elimination phase, which for all antimalarial drugs is a first-order process, and therefore has a half-life (the terminal elimination half-life).

### EPI

Expanded programme of immunizations

### First order kinetics

A reaction rate in which the rate is proportional to the concentration. In the case of drug (or malaria parasite) elimination the rate of reduction in blood concentrations at any time is proportional to the concentration at that time. The result is that a fixed fraction of the drug (or parasites) is cleared per unit time. When plotted on a semi-log scale the plot is linear, and if the vertical axis is in natural logarithms (log_e_), then the slope gives the first order rate constant (k).

### IPT

Intermittent preventive or presumptive treatment. A treatment dose of antimalarial (most studies have been with SP) is given at fixed times to treat and prevent malaria.

### IPTp

IPT in pregnancy. In HIV negative pregnant women, two doses are given – one in the second and one in the third trimester

### IPTi

IPT in infants. Treatment doses are usually given with EPI immunizations (i.e. at (2), 3, and 9 months)

### IPTc

IPT in children – sometimes called sIPT. Treatment doses are given to young children in areas with seasonal malaria during periods of peak transmission.

### IPTa

IPT in adults. Treatment doses are given to adults going to or working in areas with seasonal malaria during periods of peak transmission.

### MIC

Minimum Inhibitory Concentration (MIC) is the lowest concentration inhibiting parasite multiplication i.e. the concentration at which parasite multiplication rate per asexual cycle = 1.

### PTP

Post-treatment prophylaxis; the suppression of newly acquired malaria infections by residual antimalarial drug concentrations following a treatment dose.

### SP

Sulphadoxine-pyrimethamine
